# Chronic Kidney Disease with Mineral Bone Disorder and Vascular Calcification: An Overview

**DOI:** 10.3390/life14030418

**Published:** 2024-03-21

**Authors:** Carmine Izzo, Carmine Secondulfo, Giancarlo Bilancio, Valeria Visco, Nicola Virtuoso, Serena Migliarino, Michele Ciccarelli, Paola Di Pietro, Lucia La Mura, Antonio Damato, Albino Carrizzo, Carmine Vecchione

**Affiliations:** 1 Department of Medicine, Surgery and Dentistry, University of Salerno, Via Salvador Allende, 84081 Baronissi, Italy; csecondulfo@unisa.it (C.S.); gbilancio@unisa.it (G.B.); vvisco@unisa.it (V.V.); mciccarelli@unisa.it (M.C.); pdipietro@unisa.it (P.D.P.); acarrizzo@unisa.it (A.C.); cvecchione@unisa.it (C.V.); 2 Cardiology Unit, University Hospital “San Giovanni di Dio e Ruggi d’Aragona”, 84131 Salerno, Italy; n1virtuso@gmail.com (N.V.); serenamigliarino@gmail.com (S.M.); 3 Centro Medico Ascione Srl, 80059 Torre del Greco, Italy; lucia.lamura@hotmail.it; 4 Vascular Physiopathology Unit, IRCCS Neuromed, 86077 Pozzilli, Italy; antonio.damato85@libero.it

**Keywords:** chronic kidney disease (CKD), vascular calcification, cardiovascular disease (CVD), serum markers, imaging techniques, renal osteodystrophy

## Abstract

Chronic kidney disease (CKD) is a global health issue with a rising prevalence, affecting 697.5 million people worldwide. It imposes a substantial burden, contributing to 35.8 million disability-adjusted life years (DALYs) and 1.2 million deaths in 2017. The mortality rate for CKD has increased by 41.5% between 1990 and 2017, positioning it as a significant cause of global mortality. CKD is associated with diverse health complications, impacting cardiovascular, neurological, nutritional, and endocrine aspects. One prominent complication is CKD–mineral and bone disorder (MBD), a complex condition involving dysregulation of bone turnover, mineralization, and strength, accompanied by soft tissue and vascular calcification. Alterations in mineral metabolism, including calcium, phosphate, parathyroid hormone (PTH), vitamin D, fibroblast growth factor-23 (FGF-23), and Klotho, play pivotal roles in CKD-MBD. These disturbances, observed early in CKD, contribute to the progression of bone disorders and renal osteodystrophy (ROD). Vascular calcification (VC) is a key component of CKD-MBD, accelerated by CKD. The pathophysiology involves complex processes in vascular smooth muscle cells and the formation of calciprotein particles (CPP). VC is closely linked to cardiovascular events and mortality, emphasizing its prognostic significance. Various serum markers and imaging techniques, including lateral plain X-ray, Kauppila Score, Adragao Score, and pulse wave velocity, aid in VC detection. Additionally, pQCT provides valuable information on arterial calcifications, offering an advantage over traditional scoring systems. CKD poses a substantial global health burden, and its complications, including CKD-MBD and VC, significantly contribute to morbidity and mortality. Understanding the intricate relationships between mineral metabolism, bone disorders, and vascular calcification is crucial for effective diagnosis and therapeutic interventions.

## 1. Introduction

### 1.1. Epidemiology and Morbidity of CKD

Chronic kidney disease (CKD) is a prevalent and widespread health condition associated with a significant burden and considerable morbidity. With 697.5 million recorded cases worldwide and a global prevalence of 9.1%, CKD accounts for 35.8 million DALYs and 1.2 million deaths in 2017 [1].

Furthermore, despite being a preventable and treatable condition, CKD affects an increasing portion of the general population. Its prevalence increased by 29.3% and its all-age mortality rate grew by 41.5% between 1990 and 2017 [1]. This trend aligns with recent projections indicating that CKD is poised to become the fifth leading global cause of mortality [2].

Technological advances now enable long-term survival even after end-stage kidney disease (ESKD) through various chronic replacement therapies (RRT). The number of individuals undergoing these therapies globally has already surpassed 2.5 million and is expected to reach 5.4 million by 2030 [3].

Although readily available in wealthier countries, RRT comes with high costs that have been steadily increasing since the introduction of dialysis in the 1960s. This rise in costs contributes to the lack of access to this life-saving therapy, resulting in the premature death of up to 7.1 million people [3,4].

CKD impacts various aspects of an individual’s health. It is a recognized independent cardiovascular risk factor [5] and commonly causes anemia [6]. Additionally, it presents various neurological complications, including anxiety, sleep disturbances, motor abnormalities, depression, and cognitive impairment [7]. Due to their chronic inflammatory state, CKD patients often experience protein-energy wasting, leading to frequent malnutrition [8]. CKD also induces a complex pattern of mineral disturbances and bone disorders associated with abnormal vascular calcification and endocrine dysregulation [9,10,11,12].

This complication is referred to as chronic kidney disease–mineral and bone disorder (CKD MBD).

### 1.2. Main Features of CKD Mineral and Bone Disease

CKD MBD is a complex condition characterized by dysregulation of bone turnover, growth, mineralization, or strength, accompanied by soft tissue and vascular calcification (VC) [13,14,15]. Additionally, alterations in mineral metabolism and homeostasis are almost invariably associated with endocrine disturbances, such as secondary hyperparathyroidism and low synthesis of vitamin D [9].

While CKD MBD affects most patients with moderate and severe CKD [13], its pathophysiological changes start in the early phases of renal function impairment.

In CKD MBD, metabolic, vascular, soft tissue and endocrine–metabolic alterations are all interconnected from a pathophysiological perspective. When individually assessed, it is crucial to keep in mind the broader context to comprehend the entire picture.

## 2. Mineral Metabolism Alterations

As previously mentioned, alterations of mineral metabolism, a central aspect of CKD MBD, can manifest in the early phases of kidney disease. Some of these alterations serve as valuable laboratory markers routinely monitored in the follow-up of CKD patients [15].

### 2.1. Calcium

Serum calcium levels decrease with the progression of CKD. Maintaining adequate calcium levels is pivotal for preventing CKD MBD [9]. Both lower and higher calcium levels are associated with CKD progression and higher cardiovascular mortality [16,17], emphasizing the significance of calcium level regulation in CKD.

### 2.2. Phosphate

Phosphate, an essential element with key roles in cellular signaling and metabolism, is fundamental for bone mineralization. It is one of the main components, together with calcium, of hydroxyapatite crystals, the mineral component of bone structure. It is associated with CKD progression, end-stage kidney disease, and all-cause and cardiovascular mortality [18,19,20]. Normalizing serum phosphate in CKD is mandatory in current guidelines and clinical practice [15].

### 2.3. Parathyroid Hormone

Parathyroid hormone (PTH) is a peptide secreted by parathyroid glands, with an active form known as “intact” PTH, consisting of 84 amino acids obtained by cleavage of an inactive form [21]. It regulates calcium homeostasis stimulating calcium reuptake by kidneys, absorption by the small intestine and calcium release by the bone [22]. Its serum concentration increases as early as stage 3 CKD, with a dramatic rise in patients nearing renal replacement therapy [18,19]. Elevated PTH levels are linked to cardiovascular events and CKD progression and are an independent predictor of fractures [17,23,24]. Clinical complications include bone disruption, cardiomyopathy, soft tissue calcification and various other harmful complications. Despite being considered a uremic toxin, and its elevation being a recognized indication of treatment initiation, optimal serum PTH levels remain uncertain, and guidelines do not provide a clear indication [14,15]. Complete normalization of PTH levels is cautioned to prevent low turnover bone disease [25].

Untreated rising PTH levels inevitably lead to secondary parathyroid hyperplasia [9], reducing sensitivity to vitamin D and contributing to treatment resistance in the later phases of CKD. Patients starting RRT with higher PTH levels may exhibit a poor response even to more aggressive therapy [9,26].

### 2.4. Vitamin D

Vitamin D, more accurately described as a steroid hormone, is more than a simple vitamin. It exists in two main forms: calcidiol and calcitriol, obtained by successive hydroxylation. Of major clinical and biological interest, 25(OH) vitamin D (also known as calcidiol, or calcifediol) is produced by the liver and represents the less bioactive form but is highly circulating [27]. Via hydroxylation of its precursor, 1.25(OH) vitamin D (calcitriol), the more biologically active form is synthesized by the kidneys [28].

Although calcitriol is considered the active form of vitamin D, its serum levels are not frequently monitored, due to short half-life, lack of a standardized assay, and serum levels influenced by exogenous administration. Therefore, calcidiol is the commonly used biomarker both in clinical and research settings [29]. Calcitriol increases enteric absorption of calcium and regulates phosphate levels, along with numerous still discussed pleiotropic effects, including immune system modulation and insulin release [30,31].

Vitamin D deficiency, defined as a calcidiol serum level <20 ng/mL [32], is common in both CKD and the earlier phases of ESKD. It is associated with poor renal outcomes and increased mortality [21,28,33]. There is no universal consensus on the treatment of vitamin D deficiency, as its optimal levels to ensure bone health are yet to be defined [34]. US guidelines are prone to early intervention, while KDIGO guidelines suggest treating non-ESKD patients with nutritional vitamin D starting from CKD stage 3, reserving calcitriol and its analogs to advanced CKD with otherwise untreatable hyperparathyroidism [15,33].

### 2.5. FGF-23

Fibroblast growth factor-23 (FGF-23), a 32 kDa glycoprotein synthesized by osteoblasts and osteocytes, inhibits renal tubular phosphate reabsorption and suppresses calcitriol synthesis in the kidney [35]. Increased FGF-23 is an early alteration in CKD-MBD, and low 25(OH) vitamin D levels and high FGF-23 levels are independent predictors of poor renal outcomes, progression to renal replacement therapy, and all-cause mortality [24,36,37].

### 2.6. Klotho

Closely linked to FGF-23, Klotho is a protein with both soluble and membrane-bound states. While the latter acts as a coreceptor of FGF-23, the soluble form has pleiotropic effects including regulation of ion channels and transporters [38,39,40]. Klotho deficiency begins in the early phases of CKD [41,42], and in the later stages, Klotho resistance and deficiency lead to elevated phosphate serum levels despite high FGF-23 levels [43].

## 3. Renal Osteodystrophy, Osteoporosis, and Bone Dysregulation

The bone is composed of two main components: an organic matrix, consisting of type-I collagen, and an inorganic mineral part, compromising calcium, phosphorus and hydroxyapatite crystals [44,45]. In total, 80% of its mass is in the cortical bone, which is more compact and hard, while the remaining 20% is located in the trabecular bone, which is more fragile and spongy [46].

Osteoporosis, defined as a disease characterized by low bone mass, microarchitecture deterioration of bone tissue, bone fragility, and a consequent increase in fracture risk [47], constitutes one of the main features of CKD MBD. It is twice as prevalent in individuals with CKD compared to those with normal renal function [48], and CKD itself is an independent risk factor for osteoporosis [49]. Fractures in CKD subjects are 2–100 times more common than age-matched patients with normal renal functions [50,51], with a mortality rate threefold greater for those with CKD.

When bone structural alterations are subsequent to and caused by CKD, they are collectively known as renal osteodystrophy (ROD).

### 3.1. Pathogenesis of Renal Osteodystrophy

In recent decades, numerous advancements have been made in understanding the complex pathogenetic mechanism of ROD, although further studies are needed for a comprehensive understanding. The main alteration in ROD is an imbalance between bone formation and reabsorption, ultimately leading to bone loss [52].

Several interconnected pathways contribute to the dysregulation of bone metabolism.

Under normal conditions, a reduction in serum calcium stimulates calcium-sensing receptors (CaSR) in the parathyroid glands, leading to the release of PTH. PTH increases renal calcium reabsorption and phosphate excretion, while also enhancing the synthesis and release of calcitriol, responsible for the intestinal uptake of calcium and phosphorus [9,14,53]. With the progressive deterioration of kidney function, the kidneys become inefficient in increasing calcium reabsorption and stimulating calcitriol production. In an attempt to regulate calcium homeostasis by increasing its enteric absorption, due to the kidney’s inability to effectively increase calcitriol synthesis, this insufficient counterbalancing mechanism results in hypocalcemia, inducing a further increase in PTH release by parathyroid glands, releasing calcium from bones and weakening them further [9,14,53].

### 3.2. Bone Histology

According to international guidelines, the gold standard for ROD diagnosis is bone biopsy [15]. To facilitate the interpretation of bone biopsies, they are classified based on three main histologic parameters: bone turnover (T), mineralization (M), and volume (V) (i.e., bone mass). These three descriptors constitute the TMV system, introduced for the first time in 2006 [54].

A histological classification based on the TMV system can provide a standard nomenclature, distinguishing five different and recognizable conditions grouped in two categories of low and high bone turnover (Table 1):

1. Osteomalacia (OM), characterized by low turnover and mineralization, with normal or low bone mass (depending on severity and duration of the disease).
2. Adynamic bone disease (ABD), described as low bone turnover with normal mineralization and normal or low volume.
3. Mild hyperparathyroid-related bone disease (HPT-BD), which presents a medium turnover, normal mineralization and any bone volume.
4. Osteitis fibrosa (OF), which represents a more severe variant of the HPT-BD in a continuum specter of alterations, with high turnover, normal mineralization and normal to low volume.
5. Mixed uremic dystrophy (MUO), characterized by high turnover, abnormal mineralization and normal volume [54,55].

**Table 1:** Five different types of renal osteodystrophy (ROD). Normal values are referred to adult males of age 45–75 years [56].

ROD Types	Mineralization	Turnover	Mass
Osteomalacia (OM)	−	−	=/−
Adynamic Bone Disease (ABD)	=	−	=/−
Mild hyperparathyroid-related bone disease (HPT-BD)	=	=/+	−/=/+
Osteitis fibrosa (OF)	=	+	=/−
Mixed uremic dystrophy (MUO)	−	+	=
Normal values (adult males)	0.50 (±0.08) µ/day	16.9 (±11.15)%/year	19.8 (±6.7)%

A bone biopsy can offer unique insights into bone architecture, turnover rate, and mineralization, providing valuable indices that no other tool can reliably offer simultaneously. Despite these advantages, bone biopsy is rarely performed due to the potential risk of complications, perceived invasiveness, and, most importantly, lack of experienced pathologists capable of accurately interpreting the results [57,58].

Bone turnover is a particularly important parameter, as it is crucial for the evaluation of the most appropriate therapy of ROD (Table 2) [13].

Bone micro-indentation, a minimally invasive technique, measures bone resistance to mechanical force through a small probe, causing micro-fractures on the bone surface. This method provides information evaluated through the bone material strength index (BMSi) [59]. Despite being a promising technique, there are currently no studies validating its use for assessing bone quality in non-ESKD patients [60].

### 3.3. Serum Markers

Various blood markers have been identified to assess ROD, offering information on bone metabolism and changes more rapidly than other techniques. However, these are not always tissue-specific, and their clinical relevance may vary [61].

Of these biomarkers, FGF-23 and Klotho have already been discussed [62,63].

Alkaline phosphatase, an enzyme responsible for removing phosphate groups from nucleotides and proteins, is produced by many tissues, but primarily the liver and bone. Each produces a specific variant that can be separately identified by laboratory measurements [64]. In the bone, alkaline phosphatase stimulates the mineralization of the collagen matrix [65]. Bone-specific alkaline phosphatase (BSAP) correlates with bone formation rate. Elevated levels of BSAP and total alkaline phosphatase are associated with a higher risk of mortality and fracture in ESKD subjects [66,67]. Together with whole PTH and intact PTH, it can discriminate bone turnover patterns, identifying subjects with low or normal turnover [68,69]. Monitoring both PTH and BSAP is recommended for a more accurate assessment of bone status, rather than evaluating each alone [70].

Procollagen type-1 N-terminal propeptide (P1NP) is a byproduct of collagen type 1 production [71], and, like BSAP, is considered a bone formation marker [59]. It can be evaluated alongside osteocalcin and TRAP5b (further discussed in this paper) to enhance the diagnostic accuracy of radiological exams to identify subjects at risk for fracture [51].

Osteocalcin, a hormone produced by osteoblasts, has pleiotropic effects on bones and glucose and lipid metabolism [72,73]. These effects are strictly intertwined, as bone reabsorption and formation are energy-demanding processes [73].

Sclerostin, a protein produced by osteocytes, inhibits the differentiation and activity of osteoblasts, reducing bone formation [74,75]. Sclerostin serum levels are inversely correlated with renal function and may be implicated in low-turnover bone disease in the early CKD stages [76,77]. In CKD progression, the vicious circle of PTH resistance followed by increasing PTH levels pushes a breakthrough in peripheric tissue resistance and lead to high-turnover bone disease [78].

Tartrate-resistant acid phosphatase 5b (TRAP5b), an enzyme responsible for bone matrix catabolism, is secreted by osteoclasts. It serves as a marker of augmented osteoclastic activity and is correlated with bone turnover and the development of secondary hyperparathyroidism [79,80]. TRAP5b is an independent predictor of hip bone mineral density [51,81] and is associated with a higher risk of fracture, aiding in the identification of low- and high-turnover bone disease [82].

Of all these serum biomarkers of bone health, only alkaline phosphatase is widely available and therefore commonly utilized in daily clinical practice.

### 3.4. Diagnostic Imaging Assessment

Current guidelines recommend monitoring bone health in individuals at risk for ROD [15]. Although biopsy is the gold standard for assessing bone health, its infrequent execution has led to the development of various imaging techniques to obtain information about bone status.

Plain radiographs can identify signs of very severe CKD MBD, such as rugger-jersey vertebral aspect, tumoral calcinosis, brown tumors, or major vascular calcifications [83]. Lateral thoracic and lumbar radiographs remain the golden standard for diagnosing vertebral fractures, a crucial aspect of ROD [84].

Dual-energy X-ray absorptiometry (DXA) is perhaps the most widely used diagnostic tool for bone density (BMD), particularly for patients with stage 3–5 CKD [15]. Current data show that a decrease in BMD increases the fracture risk [85]. While DXA is widely available and offers low radiation exposure and relative cost-effectiveness, it has several important limitations. It provides a bidimensional evaluation of the bone, measuring only areal BMD without providing information about bone volume or differentiating between cortical and trabecular bones. Additionally, it cannot determine bone quality parameters regarding micro-architecture and micro-damage. DXA results can be confounded by vascular calcification (especially in the abdominal aorta), vertebral fractures and degenerative arthrosis [86,87]. To address some of these limitations, DXA can be integrated with trabecular bone score (TBS), a special software that can evaluate bone architecture in the lumbar vertebrae by characterizing trabecular bone texture [88]. TBS has proven effective in improving the correlation between DXA BMD scans and fracture risk compared to bone biopsy parameters [89,90]. The FRAX prediction model, widely used to assess the risk of 10-year fractures, can be applied to both standard DXA and TBS [91], with better results when adjusted for TBS [92]. Vertebral fracture assessment (VFA) during DXA scan can be easily performed, as it is available on most modern machines [93], but lateral X-rays are still considered the gold standard due to the low quality of images for upper thoracic vertebrae [94].

Peripheral quantitative computed tomography (pQCT) and its high-resolution derivate (HRpQCT) are low-radiation and low-cost imaging techniques [95] which are extremely useful for the evaluation of trabecular and cortical bone volumetric density [13]. pQCT accurately assesses bone volume and quality, showing a strong correlation with histologic exams [87,96,97]. HRpQCT provides greater resolution and direct quantification of cortical geometry and porosity, thickness, and trabecular parameters [98]. However, it is still limited to research settings [61]. A drawback for both pQCT and HRpQCT is their inability to evaluate central skeleton bones, and QCT is more expensive and comes with greater radiation exposure [59].

Magnetic resonance imaging is the only other available technique that can perform a three-dimensional evaluation of bone geometry and architecture without ionizing radiation. Micro-MRI, with its excellent spatial resolution, closely approximates bone histology [99,100]. However, a great limitation is the scarce availability of necessary equipment and the high cost associated with MRI.

## 4. Vascular Calcification

Vascular and soft tissue calcification are integral aspects of CKD MBD, intricately connected to ROD, particularly adynamic bone disease [101,102]. Numerous studies highlight a high prevalence of VC with increased severity and rapid progression in CKD, spanning both early stages and ESKD [103,104,105,106]. The presence and progression of VC are crucial prognostic indicators [107,108], suggesting their potential role in guiding therapeutic interventions [15,109]. While not considered a direct target in CKD [110], VC is closely correlated to cardiovascular events and cardiovascular and all-cause mortality, posing the greatest cardiovascular risk in individuals with CKD and evidence of VC [15,111].

### 4.1. Pathophysiology of Vascular Calcification in CKD MBD

Although CKD MBD is characteristic of this condition, is not solely responsible for vascular calcification. CKD acts as an accelerator of ongoing calcification processes associated with aging [112]. VC is an intricate, active process, not a mere deposition of calcium and phosphate. The transformation of vascular smooth muscle cells to a secretory phenotype plays a key role in initiating and sustaining arterial calcification [113,114].

Calcium and phosphate ions in the bloodstream can form nanocomplexes, typically removed by regulatory proteins like fetuin-A and Matrix Gla proteins. In CKD, these proteins can become oversaturated, leading to the formation of primary calciprotein particles (CPP) and amorphous calcium phosphate accumulation. These primary CPPs further convert to secondary CPPs characterized by a crystalline form [115]. Secondary CPPs create a conducive environment for the trans-differentiation of vascular smooth muscle cells into osteoblast-like cells, changing their phenotype from contractile to secretory. This transformation promotes arterial wall mineralization by producing matrix vesicles [116]. Moreover, secondary CPPs appear to stimulate inflammation and macrophage apoptosis, promoting ectopic calcification [117,118]. Conversely, some studies suggested that VC itself may act as a promoter of inflammation rather than being a consequence [119].

Vascular calcification can be categorized into two related conditions distinguished by the site of calcium crystal deposition in the arteries: medial calcification (Mönckeberg’s sclerosis) and intimal calcification. These two different manifestations of VC in CKD MBD have different characteristics and lead to different clinical manifestations. Medial calcification occurs in the absence of inflammation or lipid deposition [120,121] and is responsible for increased arterial stiffness, left ventricular hypertrophy, and subsequent heart failure [122]. In contrast, intimal calcification, pathophysiologically characterized by lipid accumulation, chronic endothelial damage [123], and inflammatory infiltration [120,124,125], is associated with ischemic events [126]. Both medial and intimal calcification occur in CKD MBD, with medial VC being more prevalent [127].

### 4.2. Serum Markers

Fetuin-A is a regulatory protein with pleiotropic effects capable of scavenging calcium-phosphate complexes, by binding its N-terminal domain [128,129]. Its role in vascular calcification is not fully understood, with clinical relevance differing in advanced and early CKD. Low serum fetuin-A correlates with rapid progression of aortic VC and major clinical adverse events [130,131]. However, recent data show no increased risk for non-ESKD subjects, despite confirming a higher risk in patients requiring RRT [132].

Matrix Gla protein (MPG) binds calcium-phosphate through negatively charged glutamate residues. Combined with fetuin-A, it initiates the CPPs (calciprotein particles) formation [129]. Carboxylation by vitamin K activates MGP, inhibiting VC. Inactivate MGP, observed in late-stage CKD, is associated with surrogate markers of mortality and VC [133,134].

CPPs, as already mentioned, can be distinguished in two sequential forms: primary CPPs, small complexes of amorphous calcium phosphate, and secondary CPPs, containing a needle-shaped crystallized complex of calcium phosphate [135]. CPPs’ serum levels increase CKD progression [136]. A recently developed in vitro assay can identify the propensity of VC formation and overall calcification by measuring the semi maximal conversion time (T50, in minutes) from primary to secondary CPPs when given additional calcium and phosphate [137]. T50 is of great clinical relevance, as it is associated with higher cardiovascular and all-cause mortality, as well as the progression of coronary artery calcification [138,139]. T50 also reflects the coexistence of other serum factors that can promote the calcification process [140].

TRAP5b is not only a bone turnover marker, but can also be prognostic for higher vascular stiffness when evaluated with BSAP [141]. TRAP5b could serve as a useful biomarker for both bone health and cardiovascular risk assessment.

Sclerostin is linked to the development of vascular calcification in non-ESKD patients more than serum phosphate levels [75,142,143]. Elevated sclerostin levels correlate with cardiovascular events, both fatal and non-fatal [144]. This association underscores the interplay between altered bone turnover and the development of vascular calcification [145].

Although not directly correlated to vascular calcification, low osteocalcin levels are associated with increased arterial stiffness and carotid atherosclerosis in CKD patients [146,147].

**Figure 1:**
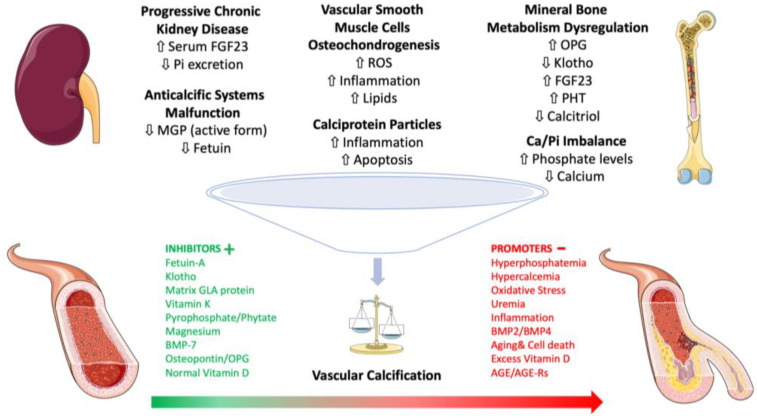
Schematic representation of the intricate network involving mediators of chronic kidney disease–mineral bone disorder (CKD–MBD) and vascular calcification. Abbreviations: CKD–MBD, chronic kidney disease–mineral and bone disorder; MGP, Matrix Gla protein; BMP, bone morphogenetic protein; AGE, advanced glycated end-products; AGE-rs, advanced glycated end-products soluble receptors; OPG, osteoprotegerin; ROS, reactive oxygen species; FGF23, fibroblast growth factor 23; PTH, parathyroid hormone; Ca, calcium; Pi, inorganic phosphate.

Vitamin K is a crucial fat-soluble vitamin known for its role in blood clotting and bone metabolism. In fact, vitamin K2 activates the Matrix Gla protein (MGP), a potent inhibitor of arterial calcification. CKD patients with higher levels of inactive, undercarboxylated MGP have a significantly increased risk of vascular calcification [148]. Vitamin K supplementation on vascular calcification progression in CKD patients reported a significant reduction in the progression of coronary artery calcification in the treated group compared to the control group [149]. The mechanisms by which vitamin K prevents vascular calcification extend beyond the activation of proteins like MGP and osteocalcin as it acts on inflammation and oxidative stress [150,151] Figure 1.

### 4.3. Diagnostic Imaging Assessment

Various imaging techniques used in clinical contexts can detect VC, computed tomography (CT), vascular and intravascular ultrasound, arteriography, and positron-emitting tomography [152,153,154]. However, current techniques cannot differentiate between intimal and medial artery calcification [155].

Current guidelines recommend a lateral plain X-ray of the abdomen to identify aortic wall calcification [15]. A semiquantitative analysis using the Kauppila Score (Table 3), since 1997 and still valid today, provides a reliable and reproducible index of VC severity and progression [156]. Kauppila score is associated with CKD progression and worsening cardiovascular parameters such as left ventricular mass, pulse pressure and left atrial volume [157].

The Adragao score (Table 4), based on X-rays of both hands and hips, is independently associated with coronary and peripheral vascular disease. It correlates with a higher risk of cardiovascular hospitalization and fatal and non-fatal cardiovascular events [109,158]. The Adragao score demonstrates a better predictive ability for hard outcomes than the Kauppila score in several studies, with extended application to non-ESKD patients [126].

Arterial stiffness, a fundamental determinant of cardiovascular complications and an independent cardiovascular risk factor [159,160,161], is associated with an increased risk of all-cause death [162]. Pulse wave velocity (PWV) is actually the gold standard for its assessment [163] due to its validation, reproducibility, standardization, and non-invasiveness. High PWV is also linked to the worsening of CKD and coronary artery calcification [164,165].

The Agatston Score can quantify the extent and severity of coronary artery calcification and heart valve calcification using computed tomography (CT). It is calculated based on the density and area of calcified lesions identified through CT imaging. In CKD patients, the Agatston Score demonstrated a strong direct correlation with increased mortality and risk of fatal and non-fatal cardiovascular events, emphasizing the prognostic significance of cardiovascular calcification in this population [166,167]. This score can also be used to monitor the rapid progression of cardiovascular calcification over time in CKD patients [168]. Despite its utility challenges existing due to factors such as arterial stiffness and arterial media calcification, the Agatston Score has been shown to underestimate coronary artery calcification in hemodialysis patients [169]. The dynamic nature of cardiovascular calcification in CKD highlights the need for vigilant monitoring using tools such as the Agatston Score.

Peripheral quantitative computed tomography (pQCT) offers valuable information beyond bone quantity and quality. It distinguishes muscle and fat areas in three-dimensional evaluation, providing a quantitative measure of arterial calcifications [170], an advantage over semi-quantitative Kauppila and Adragao scores that rely on visual assessment. Additionally, recent studies suggest an association between bone architecture assessed via high-resolution pQCT (HRpQCT) and coronary artery calcification [171]. Further research is required to establish optimal methods for using vascular calcification information provided by pQCT.

### 4.4. Therapy of Vascular Calcifications

Despite their many negative effects, there is currently no approved specific therapy for VC. Their progression can be effectively slowed in the context of the management of CKD MBD, particularly focusing on the following aims:

- Lowering serum phosphate levels: phosphate binders, preferably non-calcium-based.
- Controlling PTH levels: calcimimetic drugs, vitamin D.
- Normalizing calcium levels: calcium supplementation, vitamin D.

Other experimental therapeutic options include vitamin K, bisphosphonates, and magnesium, but current evidence, despite being encouraging, does not support their routine use in a clinical context [172].

## 5. Conclusions and Future Perspective

Chronic kidney disease is a global health challenge, affecting millions of individuals and imposing a substantial burden on healthcare systems. The rising prevalence of CKD, coupled with its diverse and severe complications, underscores the urgent need for comprehensive strategies to address this public health issue. The interconnected nature of CKD with cardiovascular, metabolic, and bone disorders highlights the complexity of its pathophysiology.

Despite the advancements in technology that allow for long-term survival after end-stage kidney disease (ESKD), the high costs associated with chronic replacement therapies pose a significant barrier to access, particularly in less affluent regions. This economic challenge contributes to the alarming number of premature deaths associated with CKD, emphasizing the critical importance of finding sustainable and affordable solutions to ensure access to life-saving therapies globally.

The focus on chronic kidney disease–mineral and bone disorder (CKD MBD) sheds light on the intricate relationships between mineral metabolism, bone health, and cardiovascular outcomes. The alterations in calcium, phosphate, parathyroid hormone, vitamin D, FGF-23, and Klotho levels in CKD MBD highlight the need for nuanced and personalized management strategies.

Furthermore, the comprehensive overview of renal osteodystrophy, osteoporosis, and bone dysregulation in CKD emphasizes the need for accurate diagnostic tools. While bone biopsy remains the gold standard, challenges such as invasiveness and lack of experienced pathologists limit its widespread use. Emerging technologies like bone micro-indentation and various serum biomarkers offer promising alternatives, yet their clinical utility requires further validation.

Vascular calcification emerges as a critical aspect of CKD MBD, with implications for cardiovascular morbidity and mortality. The intricate pathophysiology of vascular calcification, involving factors like fetuin-A, Matrix Gla protein, and calciprotein particles, underscores the need for a multifaceted approach to mitigate these complications.

Looking forward, a holistic approach to CKD prevention, early detection, and management is imperative. Investments in healthcare infrastructure, awareness campaigns, and research are essential components of a global strategy to reduce the prevalence of CKD, improve access to treatment, and enhance the quality of life for affected individuals. Additionally, ongoing research into advanced diagnostic techniques and targeted therapeutic interventions is crucial for refining our understanding of CKD and improving patient outcomes. The integration of multidisciplinary efforts from healthcare professionals, policymakers, and researchers will be pivotal in addressing the multifaceted challenges posed by CKD in the future.

## Figures and Tables

**Table 2:** Most appropriate therapy according to bone turnover status.

Turnover	Drug
Low (OM, ABD)	Anabolic agent (teriparatide, abaloparatide)
High (HPT-BD, OF, MUO)	Anti-resorptive drug (bisphosphonate, denosumab)

**Table 3:** Kauppila score: 1 point is assigned for calcification in the anterior or posterior wall of the aorta, extending for each third of each vertebra. Score ranges from 0 to 24.

Kauppila Score	Small (1/3 of Lumbar Length)	Moderate (2/3 of Lumbar Length)	Large (3/3 of Lumbar Length)
1st lumbar vertebra	1	2	3
2nd lumbar vertebra	1	2	3
3rd lumbar vertebra	1	2	3
4th lumbar vertebra	1	2	3
Total points	0–24		

**Table 4:** Adragao score: 0 points for no calcification, 1 point for presence of calcification for each quadrant of both hands and hips; values range from 0 to 8 points.

Adragao Score	Upper Quadrant	Lower Quadrant
Right hand	1	1
Left hand	1	1
Right hip	1	1
Left hip	1	1
Total points	0–8	
